# From periodontal infection to oral–systemic dysbiosis: re-framing the oral microbiome’s role in systemic health and the prophylactic potential of *Streptococcus salivarius* probiotics

**DOI:** 10.3389/fmicb.2025.1685071

**Published:** 2025-12-02

**Authors:** Francesco Di Pierro, Carlo Fornaini, Chiara Maria Palazzi, Alexander Bertuccioli, John Tagg, Nicola Zerbinati

**Affiliations:** 1 Microbiota International Clinical Society, Torino, Italy; 2 Scientific and Research Department, Velleja Research, Milano, Italy; 3 Department of Medicine and Technological Innovation, University of Insubria, Varese, Italy; 4 Department of Surgery, Medicine, Dentistry and Morphological Sciences with Transplant Surgery, Oncology and Regenerative Medicine Relevance (CHIMOMO), University of Modena and Reggio Emilia, Modena, Italy; 5 Department of Stomatology, 2nd Hospital, Shijiazhuang, Hebei, China; 6 Department of Biomolecular Sciences, University of Urbino Carlo Bo, Urbino, Italy; 7 Blis Technologies, South Dunedin, New Zealand

**Keywords:** oral microbiota, *S. salivarius* strains K12 and M18, oral-systemic axis, CRC, diabetes, obesity, rheumatoid arthritis, stroke

## Abstract

Since the late 19th century, the relationship between oral infections and systemic health has been widely debated. Recent advances in microbiome research suggest that oral microbial imbalance—dysbiosis—can contribute to systemic inflammatory and metabolic disorders. We propose that the term “oral-systemic dysbiosis” more accurately captures the complex interactions linking oral microbial disruption to systemic disease risk. This conceptual reframing moves beyond a purely infectious model toward a systems-based understanding of oral–body health connections. Furthermore, we hypothesize that oral administration of Generally Recognized as Safe (GRAS)-grade probiotic *Streptococcus salivarius*, such as strain K12, may help restore oral eubiosis and potentially reduce systemic inflammatory burden. While the hypothesis that oral probiotic maintenance may reduce the risk of systemic disorders remains to be clinically verified, defining oral–systemic dysbiosis provides a useful conceptual foundation for the exploration of integrated preventive strategies linking oral and general health.

## Introduction

The relationship between oral infections and systemic diseases has been debated for centuries. As early as ancient Greece, Hippocrates described a case in which arthritis symptoms improved following the extraction of an infected tooth (Baumgartner et al., 2008). In modern times, the concept of oral–systemic interaction was formalized through the focal infection theory. Willoughby D. Miller, in 1891, proposed that oral microorganisms could influence distant body sites (Miller, 1891), and William Hunter later linked “oral sepsis” to diverse systemic disorders including rheumatism, kidney disease, and mental depression (Hunter, 1900). Billings (1916) and others popularized the concept, leading to widespread adoption of radical treatments such as routine tooth extraction and tonsillectomy (Bynum, 2002; Kumar, 2017). However, the focal infection hypothesis also attracted criticism. While some supported it (Rosenow, 1917; Gilmer, 1916), others found the evidence unconvincing (Price, 1925; Grossman, 1960). In the 1940s, Reimann and Havens (1940) and the Journal of the American Dental Association (Eastlick, 1951) published influential reviews that rejected focal infection as a universal cause of systemic disease, favoring more conservative dental approaches. Despite this, modern microbiological and epidemiological research has revived the core concept, showing that oral dysbiosis—an imbalance within the oral microbiota—can contribute to systemic inflammation and disease. Numerous studies now link oral microbial disruption with conditions such as cardiovascular disease, diabetes, and autoimmune disorders. In 1996, Offenbacher introduced the term “Periodontal Medicine” to describe how periodontal infection and inflammation may affect extraoral health (Beck et al., 2019). Monsarrat et al. (2016) later identified 57 systemic conditions potentially related to periodontal disease, and the related term “Endodontic Medicine” was proposed to encompass associations between endodontic infections and systemic pathologies (Segura-Egea et al., 2019; Cintra et al., 2021). Beyond periodontal and endodontic tissues, chronic infections may also arise elsewhere in the oral cavity—including the tongue, palate, and dentin. Okuda and Ebihara (1998) described over 300 bacterial species inhabiting the oral ecosystem, capable of forming complex communities on teeth, gingival crevices, the tongue dorsum, and mucosal surfaces. These reservoirs may contribute to both oral and systemic inflammation, with secondary effects on tissues such as the kidneys, joints, or skin. Moreover, oral infections are often multifocal, as in endodontic–periodontal lesions, where bacterial migration occurs between root surfaces and the canal system via apical or accessory pathways (Das et al., 2020).

## Oral-systemic dysbiosis: a modern framework for understanding oral health and disease

To unify the diverse oral conditions implicated in systemic disorders under a single conceptual framework, we propose the term “oral-systemic dysbiosis” and offer the following: “Oral–systemic dysbiosis refers to a chronic, reciprocal disturbance in the equilibrium of oral microbiota that contributes causally or synergistically to systemic inflammatory or metabolic disease, mediated through microbial translocation, cytokine spill-over, or molecular mimicry.” This terminology reflects a shift away from a purely infectious perspective toward one that recognizes microbial imbalance and host–microbe interaction as central to oral-systemic pathogenesis. It encompasses dysbiotic states of the oral microbiota—regardless of anatomical site or microbial species—that may influence systemic physiology through inflammatory or translocation mechanisms. Under normal conditions, the oral microbiota maintains a state of eubiosis, in which diverse microbial communities coexist in balance with the host. Dysbiosis arises when this equilibrium is disrupted, often by environmental stressors, antibiotic exposure, or disease, leading to the overgrowth of pathogenic or inflammatory taxa (Berthouzoz et al., 2023). Such disruption has been associated with a range of systemic conditions via mechanisms that include bacterial dissemination, immune modulation, and chronic low-grade inflammation. The novelty of the oral-systemic dysbiosis framework lies not merely in terminology but in its unifying scope and mechanistic orientation. Whereas prior models such as “periodontal medicine” and “focal infection” emphasize localized infection as the initiating factor, the oral–systemic dysbiosis concept encompasses the full oral microbial ecosystem -including mucosal, salivary, and tongue-associated communities- as a dynamic participant in systemic inflammatory networks. It further recognizes the bidirectional nature of influence, whereby systemic metabolic or immune dysregulation may itself impact upon oral microbial balance. Thus, oral-systemic dysbiosis serves as an integrative construct linking microbiome ecology, immune signaling, and systemic health within a single conceptual framework.

## Relationship between oral microbiota, systemic diseases, and probiotic modulation

**Figure 1:**
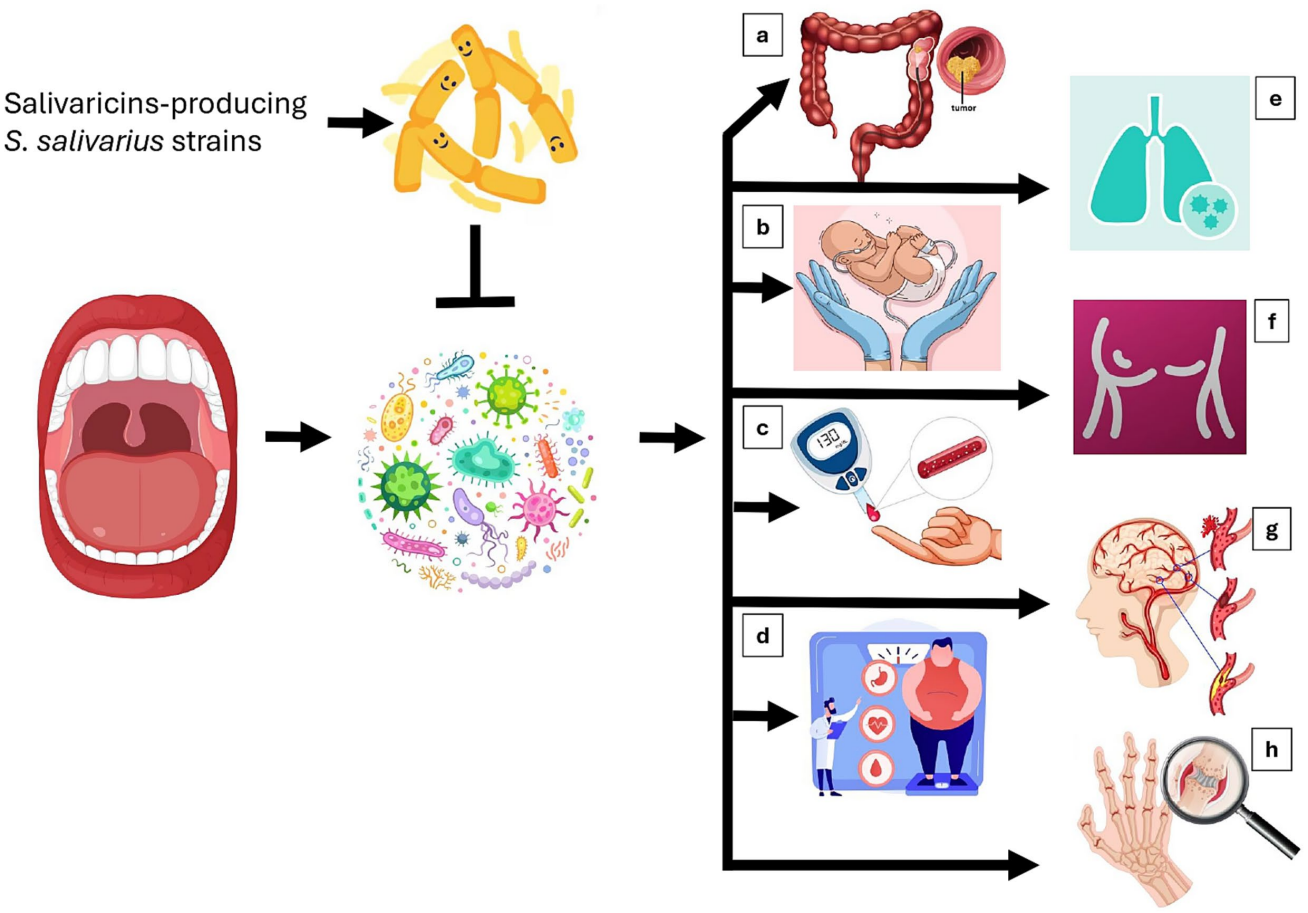
A dysbiotic oral microbiota may promote the development of systemic diseases. Restoring oral eubiosis through supplementation with *S. salivarius* probiotics could help counteract these conditions. A dysbiotic oral microbiota can act as a chronic inflammatory and metabolic driver of systemic disease. The imbalance between pathobionts and commensal microorganisms promotes epithelial barrier disruption, cytokine spillover (IL-1β, IL-6, TNF-*α*), and microbial translocation into the bloodstream or gastrointestinal tract. These processes amplify systemic inflammation, oxidative stress, and immune dysregulation. Supplementation with salivaricin-producing *S. salivarius* strains may restore oral eubiosis by competitive exclusion of pathogens, bacteriocin-mediated inhibition, modulation of mucosal immunity, and promotion of epithelial integrity, thereby mitigating downstream systemic effects. Overall, the figure illustrates the interconnected pathways by which oral dysbiosis can influence distal organs and how targeted probiotic restoration may reestablish immunometabolic balance within the oral–systemic axis. **(A)** Oncogenic modulation: Salivaricin B-producing *S. salivarius* inhibits *F. nucleatum*, an oral and colorectal oncobacterium capable of activating β-catenin signaling, promoting tumor cell adhesion, and driving chemoresistance. This antagonism may interrupt oral–gut microbial translocation and attenuate colorectal tumorigenesis. **(B)** Pregnancy and preterm birth: Bacteriocins-producing *S. salivarius* counteracts inflammatory cytokine inducers like *P. gingivalis* and other Gram-negative anaerobes linked to placental colonization. By reducing microbial invasion and systemic cytokine dissemination (IL-6, IL-8, TNF-α), it may lower the risk of preeclampsia and preterm delivery. **(C)** Autoimmune diabetes: Loss of oral–gut *S. salivarius* connectivity may favor intestinal colonization by pro-inflammatory, lipopolysaccharide-rich taxa. Its absence disrupts immune tolerance and contributes to β-cell–directed autoimmunity; conversely, probiotic restoration may support eubiosis and metabolic homeostasis. **(D)** Metabolic regulation: Swallowed *S. salivarius* can transiently colonize the small intestine, metabolizing simple carbohydrates (CHO) into lactate that fuels cross-feeding to butyrate- and propionate-producing commensals. By metabolizing CHO, *S. salivarius* produces enveloping exopolysaccharide (EP). The resulting short-chain fatty acids, derived by digestion of EP, enhance insulin sensitivity, intestinal barrier function, and host energy balance, correlating with reduced obesity risk. **(E)** Respiratory protection: Oral-derived *S. salivarius* may colonize the upper respiratory tract, suppressing Gram-negative pathogens via bacteriocins and modulating mucosal immunity. Increased *S. salivarius* abundance has been associated with attenuated inflammatory responses in cystic fibrosis and COVID-19. **(F)** Mastitis prevention: Maternal–infant transfer of *S. salivarius* allows its colonization of both the neonatal oral cavity and mammary gland. Its salivaricin-rich supernatant inhibits *S. aureus*, potentially reducing biofilm formation and lactational mastitis risk. **(G)** Cardiovascular protection: Salivaricin B-producing *S. salivarius* antagonizes *S. anginosus*, an oral pathobiont able to reach the gut, whose trimethylamine (TMA) liver metabolism generates pro-atherogenic TMAO. This inhibition may reduce stroke and cardiovascular risk. **(H)** Rheumatoid arthritis (RA) protection: *S. salivarius* suppresses *A. actinomycetemcomitans* and *P. gingivalis*, which promote citrullination of host proteins and break immune tolerance. Its presence may therefore protect against generation of anti-citrullinated protein antibodies characteristic of RA.

A growing body of literature supports associations between oral dysbiosis and systemic diseases (Figure 1). In this context, several researchers have suggested that restoration of oral eubiosis through probiotics may represent a potential prophylactic approach (Graves et al., 2019; Fan and Pedersen, 2021). For instance, *Fusobacterium nucleatum*, a common oral pathobiont, has been detected both in the oral cavity and in colorectal carcinoma (CRC) tissues of the same individuals, implicating oral–gut translocation in disease progression (Komiya et al., 2019). *Fusobacterium nucleatum* has been associated with tumor initiation, metastasis, and chemoresistance (Rubinstein et al., 2019; Yu et al., 2017). Certain *S. salivarius* strains that produce salivaricin B can inhibit the growth of *F. nucleatum* (Jang et al., 2025; Lawrence et al., 2022; Van Holm et al., 2023a), suggesting a plausible ecological counterbalance that warrants further study. During pregnancy, maternal periodontal disease has been associated with hematogenous dissemination of Gram-negative oral pathogens (*P. gingivalis*, *A. actinomycetemcomitans*, *F. nucleatum*, *P. intermedia*, *T. forsythia*) and proinflammatory cytokines (IL-1, IL-6, IL-8, TNF-*α*), contributing to adverse outcomes such as preterm birth, preeclampsia, and impaired fetal growth (Boggess, 2020). Salivaricin-producing *S. salivarius* strains, including K12 and M18, have been shown to inhibit these pathogens and to attenuate inflammatory cascades *in vitro* and in early clinical models (Yoo et al., 2020; MacDonald et al., 2021). Similarly, oral–gut interactions have been implicated in type 1 diabetes (T1D). The process of oral–gut translocation of *S. salivarius* has been linked to reduced Gram-negative bacterial abundance in the fecal microbiota, potentially influencing immune tolerance and metabolic homeostasis (Ruff et al., 2020; Kunath et al., 2022). Emerging data also suggest a role for *S. salivarius* in metabolic regulation. Through normal swallowing, *S. salivarius* reaches the small intestine, where it metabolizes simple sugars to lactate, fueling cross-feeding pathways that yield short-chain fatty acids such as propionate and butyrate (Van den Bogert et al., 2013; Zoetendal et al., 2012). These metabolites may improve insulin sensitivity and modulate host metabolism (Shimizu et al., 2025). Although these findings are preliminary, they highlight the systemic relevance of oral commensals.

In rheumatoid arthritis (RA), oral pathogens such as *P. gingivalis* and *A. actinomycetemcomitans* can generate citrullinated proteins that stimulate anti-citrullinated protein antibodies and autoimmune responses (González-Febles and Sanz, 2021; Wan Jiun et al., 2023). Experimental work has shown that oral or nasal administration of *S. salivarius* K12 can mitigate arthritis development in animal models, while healthy human subjects often display higher *S. salivarius* abundance than RA patients (Li et al., 2024). Beyond metabolic and autoimmune diseases, the presence of oral pathobionts such as *Streptococcus anginosus* in the gut has recently been linked to increased stroke risk and poorer prognosis (Tonomura et al., 2025). Given that *S. salivarius* K12 exhibits potent, salivaricin-mediated inhibition of *S. anginosus* (Burton et al., 2006a,b), this antagonism may represent an additional pathway worth investigation. Similarly, *Staphylococcus aureus*, a leading cause of mastitis (Spencer, 2008; Jiménez et al., 2015), may reach the mammary gland via retrograde flow or skin contamination (Matera et al., 2025). The supernatant of *S. salivarius* K12, rich in salivaricins, inhibits *S. aureus* proliferation (Frickmann et al., 2018). Probiotic colonization of mother–infant oral microbiota might, therefore, hypothetically reduce postpartum mastitis risk (Bogaert et al., 2023).

Finally, the lung microbiota derives largely from oral microorganisms (Huffnagle et al., 2017). In respiratory diseases—including cystic fibrosis and SARS-CoV-2 infection—greater abundance of *S. sali*var*ius* has been associated with better outcomes, and administration of strain K12 has shown protective effects through microbial and immunological mechanisms (Filkins et al., 2012; Iebba et al., 2021; Di Pierro et al., 2022; Bondareva et al., 2023).

## Discussion

The evidence summarized above supports the concept that disturbances in oral microbial ecology may contribute to systemic inflammation and disease—a phenomenon we define as oral-systemic dysbiosis. It is important to acknowledge, however, that evidence for the clinical efficacy of *S. salivarius* probiotics contains inconsistencies. Some controlled trials have reported only modest or transient colonization, and several studies have failed to demonstrate statistically significant benefits in oral or systemic health endpoints (e.g., Burton et al., 2013; Van Holm et al., 2023a,b). These inconsistencies possibly reflect variation in strain specificity, formulation stability, and host microbiome compatibility. Moreover, the *de novo* establishment of *S. salivarius* populations may depend on oral hygiene, antibiotic exposure, and host immune status. Accordingly, while current findings support biological plausibility, larger, well-controlled, and longer-term clinical studies are required to determine whether probiotic maintenance can consistently produce durable systemic effects. While many of the current associations remain correlative rather than causal, they provide a strong rationale for further mechanistic and interventional studies. We hypothesize that maintaining or restoring oral eubiosis through the use of oral-derived probiotics, particularly salivaricin-producing *S. salivarius* strains, may represent a practical approach to reducing systemic inflammatory burden. Among these, *S. salivarius* K12 is supported by extensive safety data and holds GRAS (Generally Recognized as Safe) status in the United States, with documented use in both children and adults (Burton et al., 2006a,b; Burton et al., 2010). However, we also emphasize that evidence for systemic disease prevention remains preliminary, and current findings should be interpreted as hypothesis-generating rather than clinically definitive. Variability in colonization success, individual microbiome differences, and limited long-term data all represent important constraints. Future randomized trials are needed to validate whether modulation of the oral microbiota can confer measurable systemic protection. Nevertheless, the oral-systemic dysbiosis framework encourages a more integrated understanding of health that bridges dentistry and systemic medicine. By acknowledging the interconnectedness of the oral and systemic microbiota, this model provides a rationale for exploring safe, microbiome-targeted strategies—such as GRAS-grade *S. sali*var*ius* probiotics—to promote both oral and overall well-being (Tagg et al., 2023).

## Limitations and future directions

Although the hypothesis presented is biologically plausible, several important limitations must be acknowledged. First, variability in probiotic colonization and only transient persistence are common findings that may limit the reproducibility of clinical outcomes. This phenomenon is not unique to the oral cavity but is shared by all human microbial ecosystems—including the intestinal and vaginal environments—where colonization success depends on host-related factors such as immune responsiveness, genetic background, mucosal structure, and the resident microbial composition. In the oral environment, colonization variability is further amplified by its exceptional ecological complexity. Unlike the gut, where colonization mainly occurs at luminal and mucosal interfaces, or the vaginal tract, which exhibits a relatively homogeneous niche, the oral cavity comprises numerous distinct microhabitats—including saliva, tongue, palate, teeth, supragingival and subgingival sulci, and multiple mucosal surfaces—each hosting a specific microbial community. This topographical and ecological diversity may inherently challenge the stable establishment and long-term persistence of probiotic strains such as *S. salivarius* K12. Moreover, most probiotic studies have evaluated colonization without prior modulation of the native oral microbiota. As suggested by Di Pierro et al. (2013), a short pre-treatment with chlorhexidine immediately before the first probiotic administration can transiently suppress competing bacterial populations and may facilitate *S. salivarius* colonization, improving its persistence. In addition, in line with public-health advisories and infectious-disease safety reviews, live probiotics should generally be avoided in severely immunocompromised or critically ill individuals, where rare cases of bacteremia or fungemia have been reported and high-quality safety data remain limited (Snydman, 2008). We therefore currently lack specific evidence to determine whether oral colonization strategies—such as those proposed in this work—can be safely implemented or prove therapeutically beneficial in immunocompromised subjects. Beyond these colonization-related factors, other limitations include the lack of large-scale randomized controlled trials and the absence of validated biomarkers linking restored oral eubiosis with measurable systemic outcomes. Future investigations should integrate longitudinal microbiome sequencing, cytokine and metabolomic profiling, and optimized delivery protocols to assess whether durable probiotic colonization can translate into systemic health benefits.

## Conclusion

Although causality remains to be demonstrated through longitudinal and interventional studies, the oral–systemic dysbiosis framework offers a unified conceptual model linking oral microbial imbalance to systemic pathology. It encourages a paradigm shift from infection-focused to ecology-driven thinking. *S. salivarius* K12 and M18 exemplify safe, well-characterized probiotics capable of restoring oral homeostasis and potentially moderating systemic inflammation. While clinical validation remains necessary, integration of oral microbiome modulation into preventive medicine now represents an exciting frontier in holistic health care.

## Data Availability

The original contributions presented in the study are included in the article/supplementary material, further inquiries can be directed to the corresponding author/s.
